# Notes and News

**Published:** 1901-11-16

**Authors:** 


### NOTES AND NEWS.

AMONGST the recent birthday honours conferred upon Professor Virchow is the Order of the White Eagle of Russia, conferred upon him by the Czar.

IN connection with the visit of the Duke of Cornwall and York to New South Wales, Dr. James Graham, Mayor of Sydney, has received the honour of knighthood.

WE learn that, although the Indian Government has asked for an increase of 52 in the *personnel* of the Indian Medical Service, the Secretary of State has only sanctioned an increase of half that number.

DR. GLOVER has retired from the contest for the direct representation of the profession in the General Medical Council. We are sure that the announcement of his retirement will be received with great regret by a large number of medical men.

THE treasurer of Guy's Hospital has issued a strong appeal on behalf of the Renovation and Building Fund, pointing out that Guy's Hospital was founded early in the eighteenth century, and that in view of the magnitude of the scheme for its repair and reconstruction, the expense cannot possibly be met out of the ordinary income of the hospital. The appeal is for a sum of £180,000.

IN response to the appeal issued by the Organising Committee of the Prince of Wales's Hospital Fund for London to factories, workshops, etc., the following amounts have been received :—The employees of Messrs. B. J. Hall and Co., £2 4s. 6d. ; the employees of Messrs. Horrockses, Crewdson and Co., Limited, £2 2s. ; the employees of Messrs. W. W. Rouch and Co., £1 1s. ; the employees of Messrs. Langley and Sons, £1 ; the employees of Messrs. J. Barringer and Sons, 14s. ; the employees of Messrs. George Beddow and Co., 10s. Contributions have also been received from Messrs. A. Goerz and Co., Limited, £10 10s. ; Messrs. A. R. Davis and Co., £2 2s. ; Messrs. S. and A. Calderara, £1 1s. ; Mr. J. Mansfield, £1 ; D. V. and J. C., 10s.

A FESTIVAL dinner of the Victoria Hospital for Children will be held at the Hotel Cecil on November 20th, at which Lord Roberts will preside.

THE annual dinner of the staff of the Royal Dental Hospital will be held on Saturday, November 23rd, at the Hotel Metropole, Mr. W. H. Woodruff presiding.

NEW buildings have been opened at the West of England Eye Infirmary, Exeter. They have been erected at a cost of nearly £10,000, the major portion of which has already been subscribed by generous friends of the institution.

PUBLIC opinion in France is rather difficult to move in the direction of sanitary reforms. In Paris the authorities have drawn up a scheme for improvement in house drainage. No less than 4,000 Paris landlords have refused to comply with the new regulations.

WORCESTERSHIRE is taking active steps to establish a county sanatorium for consumption. Towards this Lord Cobham has offered a fine site at Romsley on favourable terms, and Dr. Thomas Corbett is contributing £1,000 from the estate of his brother, the late Mr. John Corbett. Two schemes, for a large and a small sanatorium, are under consideration.

AT a recent conference upon the abatement of street noises, held at 1 Finsbury Circus, E.C., under the presidency of Mr. Robert Pierpoint, M.P., it was decided that the petition to the authorities should remain open during the present month, also that a deputation should be appointed to wait upon the Home Secretary requesting him to facilitate the adoption of more stringent bye-laws respecting street music, street shouting, and other unnecessary noises.

THE new Plymouth Royal Eye Infirmary has now been formally opened by Lady Mary Parker. The King has consented to be patron of the new institution, the opening of which was deferred owing to the death of Queen Victoria. The cost of site and infirmary has been somewhat under £19,000, of which £1,300 remains to be subscribed. Capital has been realised, and every means taken to provide the necessary money, without too extensive an appeal, so it is to be hoped that small difficulty will arise in removing the debt.

THE Cardiff Infirmary does its work in close touch with several mining districts, and efforts are made to enlist the support and sympathy of the miners with much success. In the Rhondda Valley alone there is ranging from Pontypridd to Treherbert a population of about 130,000, and it is felt that many of the miners are individually ready to give if some organisation worked by themselves can be found to make known what the infirmary really means, and collect their contribution for its upkeep. As it is, for the half-year ending June 30th last the infirmary received £498 from the Glamorganshire colliers and £232 from the Monmouthshire colliers, which sums, large as they are, could easily be increased without inconveniencing the donors.

